# Development of ****β****-Lactamase as a Tool for Monitoring Conditional Gene Expression by a Tetracycline-Riboswitch in *Methanosarcina acetivorans*

**DOI:** 10.1155/2014/725610

**Published:** 2014-01-20

**Authors:** Shemsi Demolli, Miriam M. Geist, Julia E. Weigand, Nicole Matschiavelli, Beatrix Suess, Michael Rother

**Affiliations:** ^1^AG RNA Biochemie, Institut für Molekulare Biowissenschaften, Johann Wolfgang Goethe-Universität Frankfurt, 60438 Frankfurt am Main, Germany; ^2^Institut für Kardiovaskuläre Regeneration, Universitätsklinikum Frankfurt, Theodor-Stern-Kai 7, 60590 Frankfurt am Main, Germany; ^3^AG Molekulare Mikrobiologie und Bioenergetik, Institut für Molekulare Biowissenschaften, Johann Wolfgang Goethe-Universität Frankfurt, 60438 Frankfurt am Main, Germany; ^4^Department für Infektiologie, Universitätsklinikum Heidelberg, Im Neuenheimer Feld 324, 69120 Heidelberg, Germany; ^5^Synthetische Biologie, Fachbereich Biologie, Technische Universität Darmstadt, 64287 Darmstadt, Germany; ^6^Institut für Mikrobiologie, Technische Universität Dresden, 01062 Dresden, Germany

## Abstract

The use of reporter gene fusions to assess cellular processes such as protein targeting and regulation of transcription or translation is established technology in archaeal, bacterial, and eukaryal genetics. Fluorescent proteins or enzymes resulting in chromogenic substrate turnover, like **β**-galactosidase, have been particularly useful for microscopic and screening purposes. However, application of such methodology is of limited use for strictly anaerobic organisms due to the requirement of molecular oxygen for chromophore formation or color development. We have developed **β**-lactamase from *Escherichia coli* (encoded by *bla*) in conjunction with the chromogenic substrate nitrocefin into a reporter system usable under anaerobic conditions for the methanogenic archaeon *Methanosarcina acetivorans*. By using a signal peptide of a putative flagellin from *M. acetivorans* and different catabolic promoters, we could demonstrate growth substrate-dependent secretion of **β**-lactamase, facilitating its use in colony screening on agar plates. Furthermore, a series of fusions comprised of a constitutive promoter and sequences encoding variants of the synthetic tetracycline-responsive riboswitch (tc-RS) was created to characterize its influence on translation initiation in *M. acetivorans*. One tc-RS variant resulted in more than 11-fold tetracycline-dependent regulation of *bla* expression, which is in the range of regulation by naturally occurring riboswitches. Thus, tc-RS fusions represent the first solely *cis*-active, that is, factor-independent system for controlled gene expression in Archaea.

## 1. Introduction

Methanogenic Archaea, a monophyletic group of strictly anaerobic archaea, are responsible for the vast majority of biologically produced methane. The process, methanogenesis, is not only highly relevant for anthropocentric concerns, such as climate change, sustainable energy strategies, waste treatment, and agriculture but also plays an essential role in the global carbon cycle because it recycles organic matter from anaerobic to aerobic environments [1] (and references therein). Methanogens convert intermediates of anaerobic biomass degradation, like H_2_+CO_2_, formate, acetate, and methylated compounds to methane via distinct yet overlapping pathways, and couple this process to energy conservation via a chemiosmotic mechanism [2, 3]. In *Methanosarcina* species, methanogenesis from methylated compounds, such as methanol or methylamines, proceeds by transfer of the methyl-group to coenzyme M (CoM) via substrate-specific methyltransferases [4] and subsequent reduction of methyl-CoM to methane [5].

Methanogens comprise one of the few genetically tractable groups among the Archaea [6] and, thus, provide outstanding model organisms for the study of this so-called “third form of life.” Despite the progress in developing tools for genetic manipulation of methanogens [7–9], many powerful techniques which are commonly applied in bacterial genetics have not yet been adapted for this group. For example, color screening of mutant libraries using *β*-galactosidase (the *lacZ* gene product) and the substrate analog X-Gal (5-bromo-4-chloro-3-indolyl-*β*-D-galactopyranoside) as a reporter system has been employed for decades in bacteria. In methanogens, both *lacZ* and *uidA* (encoding *β*-glucuronidase from *E. coli*) can also be used as reporter genes [10, 11]. However, formation of the respective chromogenic cleavage product (indigo dye) requires the presence of oxygen. Thus, for color screening of methanogenic mutants on solid media, colonies have to be exposed to oxygen requiring either replica plating or a certain degree of tolerance towards oxygen [12, 13]. To obviate the requirement of exposing strictly anaerobic *Methanosarcina* to air, we report here on a color-screenable reporter gene system active in the absence of oxygen.

Regulable gene expression systems are also powerful genetic tools. Although a variety of such systems are available for methanogenic archaea, most of them involve the use of endogenous metabolic promoters and therefore change of cultivation conditions (e.g., switch of energy substrate or nitrogen source) is required to achieve induction or repression of transcription of the gene of interest [14–17]. The only exception so far is an artificial hybrid promoter making use of the transcriptional regulator TetR and the TetR binding site* tetO*, which are part of the Tn*10*-encoded system for tetracycline (tc) resistance in *E. coli* [18]. By inserting TetR binding sites into the strong, constitutively expressing *mcrB* promoter (*mcrB*_*P*_), which controls the first gene of the *mcrBCDGA* operon encoding methyl-CoM reductase in *M. barkeri* [19], expression of genes under control of this hybrid promoter was tc-dependent in *M. acetivorans*, which is naturally tc-resistant [20]. Therefore, to achieve tc-dependent gene regulation using this system, TetR has to be coexpressed.

Riboswitches are RNA-based regulatory elements, which control a plethora of metabolic genes in bacteria frequently found in the 5′ untranslated region (UTR) of genes related to the metabolism or transport of the ligand they respond to. They are composed of an aptamer domain sensing the concentration of a cellular metabolite and an expression platform, which reads out the binding status of the aptamer domain switching gene expression ON or OFF [21]. Riboswitches were previously only predicted bioinformatically for the Archaea [22, 23]. Very recently, a fluoride-riboswitch was confirmed by genetic analysis in *Thermococcus kodakarensis* (T. J. Santangelo, personal communication). Synthetic riboswitches have been engineered for the conditional control of gene expression both in bacterial and eukaryal systems [24]. Similar to their natural counterpart, they recognize a small molecule ligand via an aptamer domain with high affinity and specificity and control the expression of the gene they reside at the level of translation initiation, pre-mRNA splicing or mRNA stability. Their independence from protein factors as well as the ability to raise synthetic binding domains (aptamers) against virtually any ligand of choice (a process called SELEX, [25, 26]) led to the widespread implementation of RNA-based regulators as control devises in the field of synthetic biology [24].

The tc-binding aptamer has been employed as synthetic riboswitch in yeast for the conditional control of gene expression [27]. It consists of three helices P1–P3, which are intercepted by single stranded regions. Multiple contacts between tc and nucleotides located in the single stranded regions result in an extremely tight binding constant in the pM range [28]. Inserted in the 5′UTR of an mRNA, the aptamer interferes with the scanning ribosome but only when its ligand is bound [29]. Further, when the aptamer is inserted close to the 5′ splice site of an intron it interferes in its ligand-bound form with splice site recognition, switching gene expression off [30]. In this example, the most prominent regulation was obtained when the complete splice site was included within the closing stem of the aptamer. Breathing of the stem in the absence of ligand allows splicing and stabilization of the aptamer upon ligand binding prevents it, making the splice site inaccessible. In the present study, we have employed the tc-RS as synthetic riboswitch for conditional control of gene expression in *M. acetivorans*. By placing the tc-RS in various distances to the ribosome binding site of the downstream gene we could observe a dynamic range of regulation.

## 2. Materials and Methods

### 2.1. Bacterial Strains and Growth Conditions

*E. coli* was grown under standard conditions [31]. *M. acetivorans* strains, listed in Table 1, were grown in high-salt medium as described [32]. Either methanol (125 mM) or trimethylamine (50 mM) served as the energy source for growth. Solid medium contained 1.5% (w/v) Bacto agar (BD, Heidelberg). For selection of the puromycin transacetylase gene (*pac*), puromycin (CalBiochem, San Diego, CA) was added from sterile, anaerobic stocks at a final concentration of 2 *μ*g mL^−1^. The purine analog 8-aza-2,6-diaminopurine (Sigma, St. Louis, MO) was added from sterile, anaerobic stocks at a final concentration of 20 *μ*g mL^−1^ for selection against the hypoxanthine phosphoribosyl transferase gene (*hpt*). Tc or doxycycline (dox) was added from sterile, anaerobic stocks at a final concentration of 200 *μ*M (or as indicated) when tc-RS-dependent *bla* translation was assessed. Growth of *M. acetivorans* was monitored photometrically at 578 nm (OD_578_).

### 2.2. Molecular Methods, Plasmid Construction and Transformation

Standard molecular methods were used for manipulation of plasmid DNA from *E. coli* [33]. All plasmids used, except for pWM321, are nonreplicating in *M. acetivorans* and are listed in Table 1. Autonomous replication in (pWM321), or integration into the chromosome of *M. acetivorans*, was selected for with puromycin. The plasmid pMR56 for insertion into the *hpt* locus of *M. acetivorans* (by homologous recombination via a truncated allele) was generated by replacing the *bla* cassette (used for selection of ampicillin resistance in *E. coli*) of pAMG48 with the kanamycin resistance cassette from pET28a(+) (Novagen). The *bla* gene from *E. coli* encoded on pBR322 was amplified by PCR without the sequence for the signal peptide and cloned into pMR56, resulting in pBlaN. There, the *bla* gene is under control of *mcrB*_*P*_ [34]. A variant where *bla* is preceded by the putative signal peptide encoding sequence from the putative *M. acetivorans* flagellin MA3061 was also constructed and designated pBlaNFSP. Plasmid pMG3 was constructed by replacing *mcrB*_*P*_ in pBlaNFSP by the ca. one kb sequence preceding *mtaC1* (MA0456), which encodes the corrinoid protein of the methanol-specific methyltransferase MT1 [35, 36]. To obtain plasmid pP0145NFSP, *mcrB*_*P*_ in pBlaNFSP was replaced by the ca. one kb sequence preceding *mtmC1 *(MA0145), which encodes the corrinoid protein of a monomethylamine-specific methyltransferase MT1 [35]. pBlaN-prom was constructed by deletion of *mcrB*_*p*_ to obtain a vector without *β*-lactamase expression used as a negative control. For promoter deletion, pBlaN was digested with BglII and NdeI, blunted and religated.

The three tc-RS encoding plasmids pBlaN_tc-RS1, pBlaN_tc-RS3, and pBlaN_tc-RS5 were constructed via a crossover PCR procedure [39]. In the first step, two asymmetric but overlapping PCR fragments were generated. pBlaN was used as template for fragment 1 encoding *mcrB*_*p*_ without the ribosomal binding site (RBS) and the 5′ end of the tc-RS. pSP64-tc-minimer [28] encoding the tc-aptamer was used for generation of fragment 2 containing the tc-RS with attached RBS. In the second step, the fragments were annealed at their overlapping region and amplified by PCR as a single fragment, using the outer primers of the first PCRs (primers pBlaN_fwd and either tc1_RBS_rev, tc2_RBS_rev, or tc3_RBS_rev, see the Supplementary Material available online at http://dx.doi.org/10.1155/2014/725610). The fragments were then cloned via BglII and NdeI into pBlaN. The other tc-RS encoding plasmids were also constructed via a crossover PCR procedure. The two overlapping PCR fragments spanning the sequence flanked by BglII and NdeI encoding the *mcrB*_*P*_-*tc*-*RS*-*RBS* fusion were generated from the precursor construct (tc-RS2 from tc-RS1, tc-RS4a from tc-RS3, and tc-RS4/4b from tc-RS4a), thereby introducing the desired nucleotide exchanges with the primers used (Supplementary Table S1).

Genomic DNA from *M. acetivorans* was isolated using a modified cetyl trimethylammonium bromide-NaCl method [40]. Unmarked chromosomal insertions in *M. acetivorans* were confirmed by Southern hybridization [41] using a probe hybridizing with the *bla* gene. All DNA sequences obtained by PCR (primers are listed in Supplementary Table S1) were confirmed by sequencing at SRD (Bad Homburg, Germany) using the BigDye Terminator Cycle Sequencing protocol (Applied Biosystems, Foster City, USA). *E. coli* was transformed by electroporation [42]. Liposome-mediated transformation of *M. acetivorans *was conducted as described [38], modified in [43] for markerless insertion of reporter constructs into the *M. acetivorans* genome [12].

### 2.3. Reporter Gene Product Activity

For qualitative detection of *β*-lactamase (Bla) activity in cultures, cell lysates, or culture supernatants, nitrocefin (CalBiochem, San Diego, USA) was added from an anaerobic sterile 1 mM stock solution (prepared according to the manufacturer's instruction) to 20 *μ*M and incubated at room temperature for 10–30 min. Culture supernatants were prepared by centrifugation of cultures at 1,500 ×g for 20 min and transfer of the supernatant into fresh anaerobic tubes. The cell sediment was lysed in deionized water (1/20 of the original culture volume) for 10 min and the lysate was brought to the volume of the original culture with high-salt medium to retain the same protein concentration as in the original culture. For Bla-dependent color development in/around colonies on agar plates, the nitrocefin stock was dispersed from a nasal spray container onto the plates after colonies of *M. acetivorans* had developed (nitrocefin is not stable for more than 2-3 h in HS medium). Cells did not loose viability by this treatment because colonies could be readily restreaked. For quantification of Bla in *M. acetivorans*, cells were harvested by centrifugation at OD_578_ of 0.4-0.5 and osmotically lysed by addition of assay buffer (100 mM potassium phosphate buffer, 1 mM EDTA, pH 7.0) containing 0.1 *μ*g mL^−1^ DNaseI. After 30 min incubation on ice, the lysate was cleared by re-centrifugation. 50 *μ*L of the cleared lysate was added to 850 *μ*L assay buffer and equilibrated for 5 min at room temperature before the assay was started by adding 100 *μ*L of a 100 *μ*M nitrocefin solution. The initial rate of nitrocefin hydrolysis was recorded for 300 seconds at 486 nm and the specific Bla activity calculated using the molar extinction coefficient for nitrocefin (*ε*_486_ = 20,500 M^−1^ cm^−1^). The values were corrected by subtracting those obtained for the strain carrying *blaN* without a promoter (pBlaN-prom), grown under the same conditions as the tc-RS-carrying strains. Protein concentration was determined by the method of Bradford [44] using bovine serum albumin as standard.

## 3. Results and Discussion

### 3.1. Synthesis and Secretion of Active *β*-Lactamase in *M. acetivorans*

**Figure 1:**
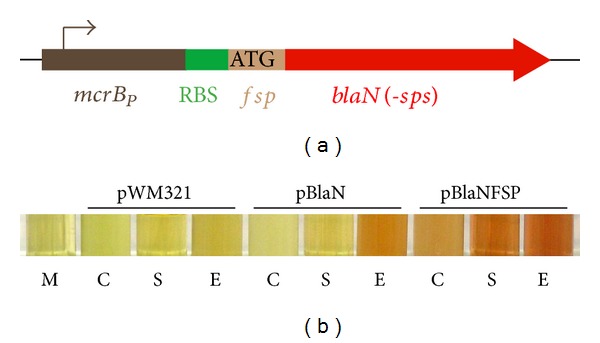
Synthesis of *β*-lactamase in *M. acetivorans*. (a) scheme of *blaN* (*bla* gene encoding *β*-lactamase and lacking the sequence for signal peptide) fusions inserted into the host chromosome; *fsp*: sequence encoding putative methanoarchaeal flagellin signal peptide; *mcrB*_*P*_: methanoarchaeal *mcrB* promoter; RBS: ribosome binding sequence; *sps*: signal peptide encoding sequence. (b) anaerobic cleavage of nitrocefin in cultures (C), culture supernatants (S), and cell lysates (E) of *M. acetivorans* strains carrying *bla* without (pBlaN) or with (pBlaNFSP) a putative archaeal signal peptide sequence on the chromosome, or the *E. coli* ampicillin resistance cassette on a self-replicating plasmid (pWM321), respectively; shown are sections of the culture tubes after 20 min of incubation at room temperature in the presence of 20 *μ*M nitrocefin.

To establish in *M. acetivorans* a chromogenic reporter system not requiring oxygen for color development, the *bla* gene from *E. coli* was chosen because a chromogenic substrate for *β*-lactamase, nitrocefin, is commercially available. Upon cleavage of the *β*-lactam ring, it undergoes a distinctive color change from yellow (*I*_max⁡_ = 390 nm at pH 7.0) to red (*I*_max⁡_ = 486 nm at pH 7.0). Furthermore, Bla is a periplasmic protein in *E. coli* allowing its potential secretion from the cell, which could facilitate its detection. To avoid interference of a plasmid-borne selection marker used for cloning in *E. coli* with our analysis, the ampicillin resistance cassette was first replaced by a kanamycin resistance cassette. Thereby, the vector backbone would not need to be removed from the chromosome after integration into the *M. acetivorans* chromosome. Using this vector (pMR56) the *bla* gene, devoid of its natural signal peptide encoding sequence, was put under control of the strong constitutive methanoarchaeal *mcrB*_*P*_ and placed onto the *M. acetivorans* chromosome (Figure 1(a)). As cleavage of an exogenous chromogenic Bla substrate and, thus, color development, would require either cell lysis or Bla excretion, a *bla* variant encoding a putative protein translocation signal peptide (from MA3061, pBlaNFSP) was also created and placed onto the *M. acetivorans* chromosome (Figure 1(a)). Both strains synthesized active Bla as evidenced by nitrocefin cleavage when cell lysates were assayed (Figure 1(b), “E”). While in the strain synthesizing Bla without a signal peptide (pBlaN) no nitrocefin cleavage was observed in the culture or the culture supernatant, color developed in the untreated culture and in spent medium when Bla contained an archaeal putative signal peptide (pBlaNFSP). Together, these data demonstrate that (i) Bla activity in *M. acetivorans* is sufficiently stable in high-salt medium to allow visual detection, that (ii) the amino acid sequence employed ((M)WNTFSKDEKGFTG) is a functional protein export signal in *M. acetivorans*, and that (iii) Bla of *E. coli* is translocated over the *M. acetivorans* cytoplasmic membrane in a functional form. Color development became visible after 10–30 min of incubation. However, prolonged incubation (>3 h) led to moderate color development in plain medium indicating abiotic cleavage of the *β*-lactam of nitrocefin (data not shown), which is possibly due to the highly reducing conditions of the medium. Furthermore, overnight incubation led to complete loss of any color indicating that nitrocefin is hydrolyzed during this long incubation. Therefore, nitrocefin cannot be included in the medium during growth of the organism.

### 3.2. Growth Substrate-Dependent Synthesis of *β*-Lactamase in *M. acetivorans*

**Figure 2:**
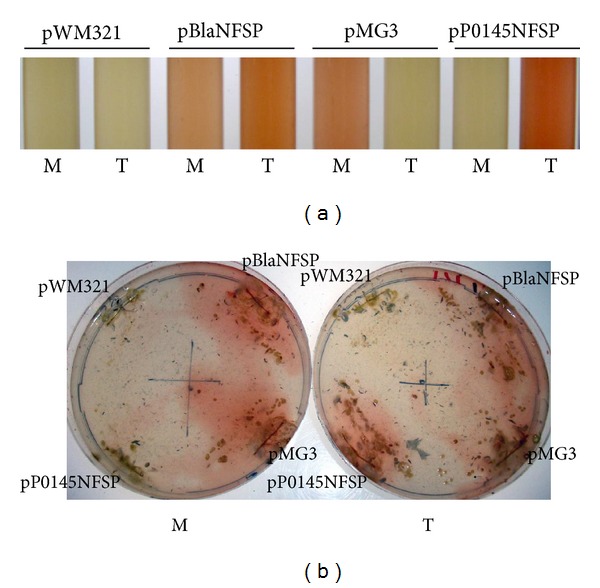
Growth substrate-dependent nitrocefin cleavage. Cultures of *M. acetivorans* transformed with pWM321 (negative control), pBlaNFSP (positive control, *mcrB*_*p*_-*fsp*-*blaN*), pMG3 (*mtaC1*_*P*_-*fsp*-*blaN*), and pP0145NFSP (*mtmC1*_*P*_-*fsp*-*blaN*) were grown on methanol (M) or trimethylamine (T) and assayed for Bla (see Materials and Methods) in liquid culture (a) or after streaking and growth on agar plates containing the respective substrate (b); shown are sections of the culture tubes (a) and plates (b) after 20 min of incubation at room temperature in the presence of nitrocefin.

To achieve growth substrate-dependent reporter synthesis in *M. acetivorans*, the strong constitutive *mcrB*_*P*_ preceding *bla* was exchanged with *mtaC1*_*P*_ (pMG3), which leads to high expression of the genes controlled by it in the presence of methanol [19], and with *mtmC1*_*P*_ (p0145NFSP), which should lead to methylamine-dependent expression of the gene controlled by it. The plasmids were integrated into the *M. acetivorans* C2A chromosome and the resulting strains examined for Bla synthesis. Methanol- or methylamine-dependent synthesis and excretion of Bla could indeed be observed in liquid cultures of both strains (Figure 2(a)). When both strains were streaked on agar plates containing either of the growth substrates, only colonies of the strain harboring p0145NFSP clearly regulated *bla* expression (Figure 2(b)). The strain harboring pMG3 developed color under both conditions (Figure 2(b)), which is due to fact that in the agar, some energy source is present that is metabolized via the methanol utilization pathway [19] leading to induction of the methanol utilization machinery while growing on methylamine-containing agar plates. A plating procedure for growth of *M. acetivorans* on an agar-free surface, which involves filtration of cells onto nitrocellulose filters and incubating them on media-soaked filter paper, has been established [19] and would have to be used in conjunction with pMG3 to obtain colonies not producing Bla in the absence of methanol. Presently, five *trans*-acting factors, MsrA, MsrB, MsrC, MsrD, and MsrE, are known to regulate expression of the genes encoding three methanol-specific MT1 isoforms MtaCB1, MtaCB2, and MtaCB3 on the level of transcription initiation [45]. The methanol-specific MT1 isoforms are also regulated at the posttranscriptional level [46] but the factor(s) involved are unknown. Transcriptome analyses demonstrated substrate-dependent regulation of mono-, di-, and trimethylamine-specific MT1 isoforms in *M. mazei *Gö1, *Methanococcoides burtonii*, and *M. acetivorans*, Mtm [47–49], but factors involved are also not known. Thus, both fusion constructs presented herein will be suitable tools to identify factors affecting methanol- and methylamine-dependent regulation by random loss-of-function mutagenesis [34]. Because the *mtaC1*_*P*_-*fsp*-*bla* fusion (pMG3) is expressed on agar lacking methanol, its use (at least under these conditions) will be restricted to identify mutants not expressing *bla*, that is, mutants that have lost (an) transcriptional activator(s) of *mtaC1*_*P*_-dependent expression. As for methylamine-dependent regulation, both negative and positive effectors of *mtmC1*_*P*_ expression can be sought for using the *mtmC1*_*P*_-*fsp*-*bla* fusion (p0145NFsP) and standard agar media. Such efforts are underway in our laboratory and will be reported elsewhere.

Archaea, like bacteria, employ two pathways for protein translocation, the Sec and the Tat (twin arginine motif) pathway [50]. While the substrates of the former are unfolded during the process, the latter translocates (at least partially) mature proteins. Since Bla from *E. coli* is a periplasmic protein and secreted via the Sec pathway, we tagged it with an N-terminal signal peptide from a putative flagellin encoded in the *M. acetivorans* genome (MA3061), assuming that it would be translocated by the corresponding pathway, despite the fact that the organism is nonmotile [51]. In *Methanococcus* species, the N-terminal signal peptides of the preflagellins are cleaved off by the type IV prepilin peptidase FlaK during translocation over the membrane [52, 53]. Therefore, any protein susceptible to the Sec pathway could be produced and secreted from *M. acetivorans* (without the signal peptide), which could greatly facilitate purification efforts. For Archaea, such a system for protein overproduction combined with secretion has so far been established only in the hyperthermophilic *Thermococcus kodakarensis* [54].

### 3.3. Developing the tc-Responsive Riboswitch for Conditional Gene Expression in *M. acetivorans*

**Figure 3:**
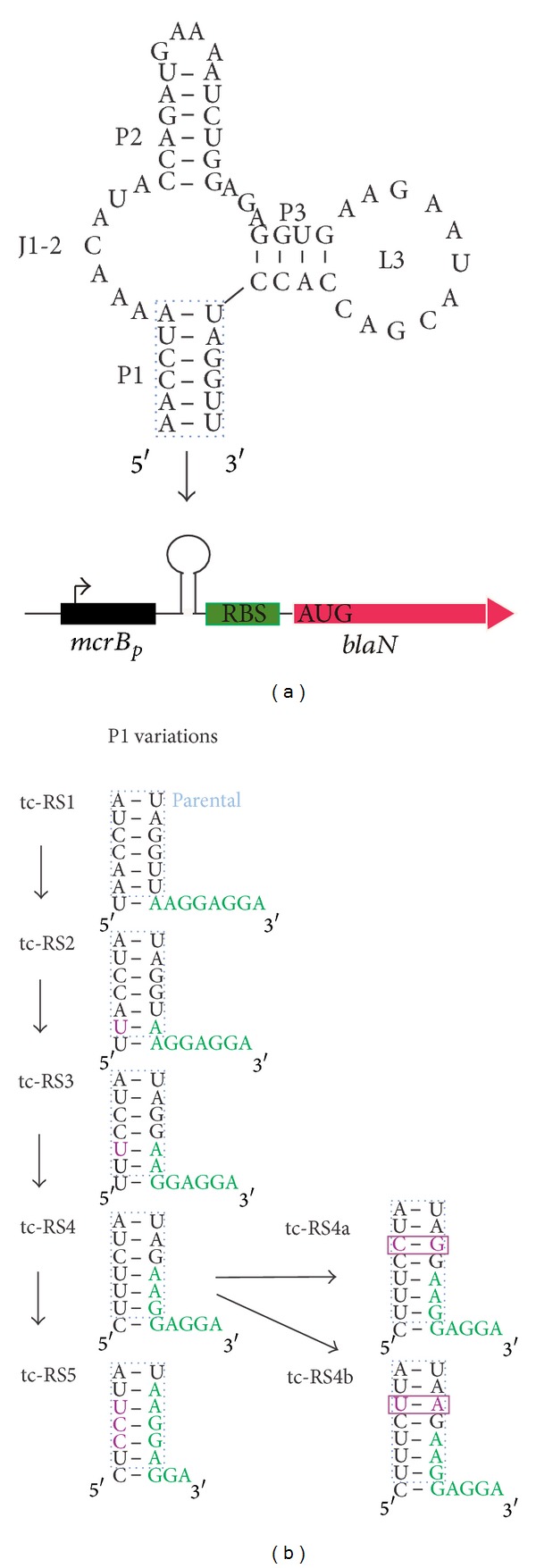
tc-RS-regulated translation of *bla *mRNA. Left: scheme of tc-RS encoding *blaN* fusions inserted into the *M. acetivorans* chromosome; right: tc-RS variants analyzed in this study (tc-RS1 to tc-RS5); for simplicity, only the closing stem P1 and the adjacent RBS (in green) are shown and bases changed from the precursor construct are indicated in violet; *mcrB*_*P*_: methanoarchaeal *mcrB* promoter.

Regulation of mRNA translation by naturally occurring riboswitches has not been reported for Archaea. A tc-RS, when fused to a leaderless reporter mRNA in the halophilic archaeon *Haloferax volcanii*, totally inhibited translation even in the absence of tc, which was taken as evidence that it forms a too stable structure in the high-salt cytoplasm of that organism [55]. To test whether tc-RS can be used in methanoarchaea as a means to control gene expression in *cis *(Figure 3(a)), its encoding sequence was inserted into *mcrB*_*P*_ of the *mcrB*_*P*_-*blaN* fusion (pBlaN), immediately upstream of the RBS (Figure 3(b), tc-RS1), and the construct placed onto the *M. acetivorans* chromosome. Bla activity could now be used as a measure for tc-dependent translation of *bla* mRNA (Table 2). Compared to the control without tc-RS (pBlaN), *bla* mRNA translation in the absence of tc was increased by ca. 2.5-fold, which is not uncommon and probably due to increased mRNA stability in the presence of a structured RNA element at the 5′ end of a bacterial mRNA [56]. In the presence of tc, Bla activity was reduced by a factor of 2.6, indicating that the tc-bound aptamer interferes with translation of the *bla* mRNA, probably by sterical hindrance of the recognition of the RBS. This is similar to the regulation observed for pre-mRNA splicing by the same riboswitch [30]. There, the aptamer was inserted next to the 5′ splice site. Regulation of splicing was strongly dependent on the distance of the aptamer from the splice site and the stability of the closing stem. The fact that best regulation of pre-mRNA splicing was obtained when the splice site was completely integrated within the closing stem [30] prompted us to successively move the RBS preceding *bla* into the closing stem of the tc-RS, giving rise to the constructs tc-RS2 to tc-RS5 with 2, 3, 4, and 6 nucleotides included, respectively (Figure 3(b)). The pairing bases were changed accordingly (Figure 3(b), marked in violet). Inclusion of the RBS into the closing stem by 1, 2, and 3 nucleotides (tc-RS1, tc-RS2, and tc-RS3) successively reduced Bla activity (104 ± 2, 71 ± 2 and 43 ± 5 mU mg^−1^, resp.; see Table 2) indicating a generally somewhat diminished access of the ribosome to its binding site. Corroborating this notion was the observation made using tc-RS5 where the RBS was moved by six nucleotides into the closing stem of tc-RS, which almost completely abolished Bla activity (Table 2). Apparently, the RNA element completely blocked access of the ribosome from this construct. Furthermore, constructs tc-RS2 and tc-RS3 did not show an improved regulation compared to tc-RS1 (1.8- and 2.7-fold). Construct tc-RS4 with four nucleotides of the RBS within the closing stem was exceptional because it resulted in a 10-fold increase in Bla activity as compared to *mcrB*_*p*_ (pBlaN). This may be explained by the fact that this mutant lacks the two neighboring CG base pairs in the centre of P1 present in all other constructs resulting in destabilization of the closing stem (ΔG of P1 = −4.4 kcal/mol, Table 2). Despite the high Bla activity in the absence of the ligand, presence of tc significantly decreased Bla activity to a level compared to the other constructs resulting in a more than 11-fold regulation of gene expression.

Based on tc-RS4, two further constructs were designed. In tc-RS4a and tc-RS4b, the closing stem was elongated by one additional base pair (tc-RS4a: GC, tc-RS4b: AU). However, the additional base pair is accompanied by a decrease in Bla activity irrespective of its stability (21 ± 2 and 14 ± 2 mU mg^−1^ for tc-RS4a and tc-RS4b, resp.). Regulation in response to tc was 2.6- and 2.0-fold, respectively, which is in the range of the constructs tc-RS1–tc-RS3. Further stabilization by increasing the length of the stem to nine base pairs resulted in even further reduction of Bla synthesis and complete loss of tc-dependent regulation (data not shown).

Of all tc-RS variants fused to *mcrB*_*P*_ and investigated here six resulted in tc-dependent expression of *bla*. Furthermore, Bla activity in the absence of tc and the dynamic range of tc-dependent regulation varied in the constructs with respect to all parameters tested (stem length and stability, location of the RBS with respect to the stem, Table 2). The data suggest that a lower stability of the closing stem (no neighboring GC base pairs) and the partial inclusion of the RBS into the stem is beneficial for both Bla activity and regulation. Of the variants tested here tc-RS4 apparently has the optimal characteristics leading to a level of regulation (almost 12-fold), which is well within the range of naturally occurring riboswitches controlling mRNA translation.

### 3.4. The tc-Responsive Riboswitch Is Ligand-Specific and Dose-Dependent *In Vivo*

As *in vivo* regulation of the tc-RS4 variant was highest its ligand specificity and dynamics of regulation were assessed further. The tc-RS is highly specific for tc and the lack of a hydroxyl group at position R_6_ *β*, resulting in dox, reduces aptamer binding by more than two orders of magnitude (0.8 nM for tc and 118 nM for dox) [28]. To address the specificity, cells harboring the tc-RS4 construct were grown in the absence or presence of 200 *μ*M dox before Bla activity was assessed. While presence of tc in the medium leads to a ca. 12-fold reduction in Bla activity, dox had no effect on Bla activity; in the absence of antibiotic 340 ± 11 mU mg^−1^, and in the presence of dox 383 ± 33 mU mg^−1^ were determined. This observation is in full accordance with the respective dissociation constants of tc-RS determined *in vitro*. At the concentration used (200 *μ*M dox), formation of the ligand-stabilized aptamer does not occur due to this lower affinity and thus *bla* translation is not repressed. It is very unlikely that the effect observed is due to a less efficient uptake of dox by *M. acetivorans* because this compound efficiently mediates *in vivo* derepression of the artificial *mcrB*_*P*_ *(tetO)*/TetR system in this organism [20].

**Figure 4:**
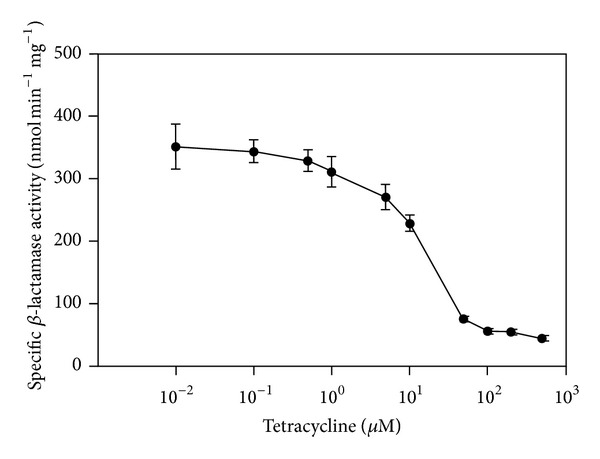
Dose-dependence of tc-RS4-mediated repression of *bla* mRNA translation. *M. acetivorans* carrying the *mcrB*_*p*_-*tc*-*RS4*-*blaN* construct on the chromosome was cultivated in the presence of varying amounts of tc before specific Bla activity was assessed.

To investigate the dynamics of riboswitch regulation, cells harboring the tc-RS4 fusion were grown in the presence of increasing amounts of tc before Bla activity was determined (Figure 4). A significant reduction in Bla activity could be observed already at 7 *μ*M tc. Half-maximal reduction of *bla* translation was observed at ca. 14 *μ*M tc and between 50 *μ*M and 100 *μ*M tc, maximal repression of translation was achieved (Figure 4). These data are in accordance with the concentration required for complete repression in yeast [27]. Interestingly, it is also in the same range as the tc concentration required to fully derepress the artificial *mcrB*_*P*_ *(tetO)*/TetR system *in vivo* (ca. 70 *μ*M) [20].

## 4. Conclusions

This work illustrates that *β*-lactamase together with nitrocefin can be employed in *Methanosarcina* as a chromogenic reporter under strictly anaerobic conditions. The *bla* gene may be fused to any promoter allowing convenient monitoring of its expression. A secreted reporter may further aid screening purposes, like identification of *trans*-active mutations, on solid media. Further, establishing the solely *cis*-active tc-responsive riboswitch in *Methanosarcina* now allows conditional control of gene expression without the need of an additional factor and represents the first synthetic riboswitch described in Archaea.

## Supplementary Material

Supplementary Table S1: Primers used in this study.

## Figures and Tables

**Table 1:** Plasmids and strains used in this study.

Plasmid	Construction^a^/description/relevant genotype	Reference/source
pBR322	Source of *bla* gene	[37]
pET28a(+)	Source of *kan* ^*R*^ marker	Novagen
pAMG46	For integration into *M. acetivorans hpt *locus; it contains *mtaC*1_*P*_	[19]
pAMG48	For integration into *M. acetivorans hpt *locus; it contains *mcrB*_*P*_	Guss and Metcalf, unpublished
pWM321	*E. coli*/*M. acetivorans* shuttle vector	[38]
pMR56	1,176 kb AlwNI/XmnI *kan* ^*R*^ fragment from pET28a(+) blunted with Klenow, ligated into AhdI/ApaLI restricted and then blunted pAMG48	This study
pBlaN	806 bp NdeI/NotI restricted *blaN* PCR fragment (primers 1 and 3) ligated into NdeI/NotI restricted pMR56; *bla* without signal peptide encoding sequence (*blaN*)	This study
pBlaNFSP	845 bp NdeI/NotI restricted blaNFSP PCR fragment (primers 2 and 3) ligated into NdeI/NotI restricted pMR56; *bla* signal peptide encoding sequence of MA3061	This study
pMG3	1,007 bp *mtaC*1_*P*_ NdeI/BglII fragment from pAMG46 ligated into NdeI/BglII pBlaNFSP	This study
p0145NFSP	1,424 bp NdeI/BglII restricted *mtmC*1_*P*_ PCR fragment (primers 4 and 5) ligated into NdeI/BglII restricted, pBlaNFSP	This study
pSP64-tc-minimer	Source of tc-RS encoding sequence	[28]
pBlaN-prom	*mc* *rB*_*P*_ in pBlaN deleted; used as negative control	This study
pBlaN_tcRS1	PCR fragment encoding tc-RS1 in the *mcrB*_*P*_ 5′UTR ligated into BlgII/NdeI restricted pBlaN	This study
pBlaN_tcRS2	PCR fragment encoding tc-RS2 in the *mcrB*_*P*_ 5′UTR ligated into BlgII/NdeI restricted pBlaN	This study
pBlaN_tcRS3	PCR fragment encoding tc-RS3 in the *mcrB*_*P*_ 5′UTR ligated into BlgII/NdeI restricted pBlaN	This study
pBlaN_tcRS4	PCR fragment encoding tc-RS4 in the *mcrB*_*P*_ 5′UTR ligated into BlgII/NdeI restricted pBlaN	This study
pBlaN_tcRS4a	PCR fragment encoding tc-RS4a in the *mcrB*_*P*_ 5′UTR ligated into BlgII/NdeI restricted pBlaN	This study
pBlaN_tcRS4b	PCR fragment encoding tc-RS2 in the *mcrB*_*P*_ 5′UTR ligated into BlgII/NdeI restricted pBlaN	This study
pBlaN_tcRS5	PCR fragment encoding tc-RS5 in the *mcrB*_*P*_ 5′UTR ligated into BlgII/NdeI restricted pBlaN	This study

^a^DNA sequences and maps of all plasmids are available upon request; primers used are listed in Supplementary Table S1.

**Table 2:** tc-dependent Bla activity in *M. acetivorans* strains carrying *mcrB_P_*-*tc*-*RS*-*blaN* gene fusions.

tc-RS construct	Bla activity^a^		-fold regulation^b^	P1 length (bp)	RBS bases in P1	P1 stability Δ*G* (kcal mol^−1^)^c^
	−tc	+tc				
*mc* *rB*_*P*_	36 ± 6	39 ± 4	—	—	—	—
tc-RS1	104 ± 2	40 ± 2	2.6	7	1	−5.6
tc-RS2	71 ± 2	40 ± 3	1.8	7	2	−5.6
tc-RS3	43 ± 5	16 ± 2	2.7	7	3	−5.1
tc-RS4	340 ± 10	29 ± 9	11.6	7	4	−4.4
tc-RS4a	21 ± 2	8 ± 1	2.6	8	4	−7.7
tc-RS4b	14 ± 2	7 ± 1	2.0	8	4	−7.7
tc-RS5	1.5 ± 0.4	n.d.	—	7	6	−7.1

^a^Values are given in mU (nmol min^−1^) mg^−1^ and are averages of three independent cultures grown in the absence (−) or presence (+) of 200 *μ*M tc; ± denotes the standard deviation; all experiments were reproduced at least once.

^b^Values (−tc/+tc) were corrected by substracting those of the negative control (-prom).

n.d.: below detection limit of the assay.

^c^Stability was calculated using mfold (http://mfold.rna.albany.edu/).
