# Supplementary material for: Development of β-Lactamase as a Tool for Monitoring Conditional Gene Expression by a Tetracycline-Riboswitch in Methanosarcina acetivorans
Source: Archaea. 2014 Jan 20;2014:725610. doi: 10.1155/2014/725610 (PMC3942078; doi:10.1155/2014/725610)
Supplement: Supplementary file 1 — Supplementary Table S1: Primers used in this study. [file 725610.f1.pdf]

Electronic Supplementary Material

Development of β-lactamase as a tool for monitoring conditional gene expression by a tetracycline-riboswitch in *Methanosarcina acetivorans*

Shemsi Demolli[1], Miriam M. Geist[2], Julia E. Weigand[1,3], Nicole Matschiavelli[4], Beatrix Suess[1,3], and Michael Rother[2,4]*

[1]AG RNA Biochemie and [2]AG Molekulare Mikrobiologie und Bioenergetik, Institut für Molekulare Biowissenschaften, Johann Wolfgang Goethe-Universität Frankfurt; [3]Synthetische Biologie, Fachbereich Biologie, Technische Universität Darmstadt, [4]Institut für Mikrobiologie, Technische Universität Dresden

*Corresponding Author: Michael Rother
Institut für Mikrobiologie
Technische Universität Dresden
D-01062 Dresden, Germany
Phone: ++49-351-46342611
Fax: ++49-351-46337715
e-mail: michael.rother@tu-dresden.de

Supplementary Table S1: Primers used in this study

No.	Name	Sequence (5'→ 3')	Use
1	oblaN/5'	ccatggcatatgCACCCAGAAACGCTGGTGAAAG	Amplification of *bla* without signal peptide sequence
2	oblaNFSP	ccatggcatATGTGGAACACATTTTCAAAAGAT GAAAAAGGATTCACTGGACACCCAGAAA-CGCTGGTGAAAG	Amplification of *bla* with signal peptide sequence from MA3061
3	obla/3'	ctgcaggcggccgcTTACCAATGCTTAATCAGTGA GGC	Amplification of *bla*
4	oP0145/5'	ccatggagatctGGCTACAGCCTGAATG	Amplification of ca. 1kb promoter region preceding MA0145
5	oP0145/3'	ccatggcataTgTTTCCCGGTTTTTCGG	Amplification of ca. 1kb promoter region preceding MA0145
6	pBlaN_fwd	CAAGCTTTGCTTTCGTATCC	Construction of tc-RS variants
7	pBlaN_rev	TGTTGAGATCCAGTTCGATG	Construction of tc-RS variants
8	tc1_prom_rev	CTTTCATCTGGTATGTTTTAGGTTATTTATT AAAATCATTTTGGGAC	Construction of tc-RS1

9	prom_tc1_fwd	GTCCCAAAATGATTTTAATAAATAACCTAA AACATACCAGATGAAAG	Construction of tc-RS1
10	tc1_RBS_rev	CGACGTCATATGAATTTCCTCCTTAACCTA GGTGGTCGTATTCTTC	Construction of tc-RS1
11	tc11_fwd	CAGTCCCAAAATGATTTTAATAAATTAACC TAAAACATACCAGATG	Construction of tc-RS2
12	tc11_rev	CATCTGGTATGTTTTAGGTTAATTTATTAAA ATCATTTTGGGACTG	Construction of tc-RS2
13	tc2_prom_rev	CTTTCATCTGGTATGTTTTAGGAAATTTATT AAAATCATTTTGGGAC	Construction of tc-RS3
14	prom_tc2_fwd	GTCCCAAAATGATTTTAATAAATTTCCTAA AACATACCAGATGAAAG	Construction of tc-RS3
15	tc2_RBS_rev	CGACGTCATATGAATTTCCTCCTTCCTAGGT GGTCGTATTCTTC	Construction of tc-RS3
16	tc6_fwd	CAGATGAAAGTCTGGAGAGGTGAAGAATA CGACCACCTAGAAGGAG	Construction of tc-RS4
17	tc6_rev	CCTCTCCAGACTTTCATCTGGTATGTTTTAG AAAGTTTATTAAAATC	Construction of tc-RS4
18	prom_tc5_fwd	GTCCCAAAATGATTTTAATAAACTTTCCTA AAACATACCAGATG	Construction of tc-RS4a
19	tc5_prom_rev	CATCTGGTATGTTTTAGGAAAGTTTATTAA AATCATTTTGGGAC	Construction of tc-RS4a
20	tc9_fwd	CATACCAGATGAAAGTCTGGAGAGGTGAA GAATACGACCACCTAAGAAGGAG	Construction of tc-RS4b
21	tc9_rev	CTCTCCAGACTTTCATCTGGTATGTTTTAAG AAAGTTTATTAAAATC	Construction of tc-RS4b
22	tc3_prom_rev	GACTTTCATCTGGTATGTTTTAAGGAGTTTA TTAAAATCATTTTGGGACTG	Construction of tc-RS5
23	prom_tc3_fwd	CAGTCCCAAAATGATTTTAATAAACTCCTT AAAACATACCAGATGAAAGTC	Construction of tc-RS5
24	tc3_RBS_rev	CGACGTCATATGAATTTCCTCCTTAGGTGG TCGTATTCTTCAC	Construction of tc-RS5
